# Editorial

**Published:** 1859-07

**Authors:** 


### Editorial.

### THE MEETING AT NIAGARA.

The next meeting of the "American Dental Convention" will be a very important one, and it is to be hoped that it will be well attended. The place of meeting is central, between East and West, and is a most pleasant retreat from the burning sun of the South ; there will, therefore, be less excuse for non-attendance than formerly. We do not see why the Niagara meeting may not be made more profitable than any preceeding one. It is true, the attractiveness of the place may lead some to seek pleasure rather than science, but we trust the majestic spirit of Niagara will suppress all disposition to trifle.

As to the formation of a delegated National Society, we have only to say that the idea, if properly carried out, is a good one. Such an organization is not at all incompatible with the continuance of the Convention, or, if it is, we are opposed to it, at the present time. The "call" is issued at such a late day, that we fear many local societies will find it impracticable to appoint delegates. At any rate, some of them must suffer the inconvenience of called meetings, or go unrepresented. If the representation is not general, it would only be generous to take the preliminary steps toward, but postpone a final organization for another year.

W.

### TO CORRESPONDENTS AND CONTRIBUTORS.

We are pleased and desirous at all times to receive contributions from the pens of our professional brethren for the *Register*, upon subjects pertaining to dental science. Indeed, to the aid thus received, does the *Register* owe much, for its success, and the value that may be attached to it. As every reader of the *Register* very well knows, we do not require, nor even wish that any article shall be in harmony with our own views, to gain admission to its pages. We are glad to have every one promulgate his own views, when they are in accordance with common sense, or when there is a respectable portion of the profession entertaining the same opinion.

Now, while we do not care *what* our brethren write, while it comes within the range of these provisions, we are desirous that some regard be given to the literary character of the productions. While there are in the dental profession some fine writers, there are some to whom this term can in no sense be applied. We occasionally receive articles for publication, from which it is impossible to gather the writer's ideas. Sometimes the language is badly put together; in other instances, there is entirely too much language, a verbosity that covers up the ideas wholly, or so obscures them, that they can not be perfectly seen. We should always remember that language is only the vehicle of thoughts; there should be a definite idea in every sentence.

Occasionally the orthography and punctuation are not of the most approved kind; indeed, some do not pretend to punctuate at all, and request us to punctuate their composition. Well, we have tried to do so, sometimes; but in future will not attempt it, unless the writer sends the ideas along with the composition. It is rather difficult to punctuate without a knowledge of the meaning of the writer. There is no excuse for bad orthography.

The most of our correspondents prepare their manuscript well, but some prepare it *otherwise*. This latter class will please send in their communications a month or two in advance of publication, that there may be ample time for re-writing.

T.

### THE AMERICAN JOURNAL.

The April number of this pioneer journal gives evidence that its "youth is renewed like the eagle's." Its original department is much more interesting than usual, and we are told by the editors that they have made definite arrangements with prominent dentists, by which this department will be fully sustained. We can testify from our own short experience, that they have adopted the only reliable mode of securing original matter of the kind and to the extent that the profession requires. We are glad to see this evidence of increased vitality; for, though we disapprove of some things which the *Journal* says and does, yet it has no more sincere well-wisher than its "annonymous friend." W.

### AMALGAM WORK—DR. HUNTER.

Dr. Hunter's article in the present number of the *Register*, starts out by referring to some observations which we made editorially in the March and June numbers of the *Register*, upon amalgam work, as put up by Dr. Slayton. At the time the remarks were made in the March number, our knowledge of the work was very limited, and we spoke of the matter as it then presented itself. The object aimed at, and the appearance of the work was given, and our own views of the work at that time, predicated upon its appearance, and the claims set up for it. We at that time had no experience with the work, and could predicate no remarks upon that basis. At that time we did not give the name of the inventor, nor the mode of doing the work, because we did not think that either was important for that notice, and we were not fully posted in regard to that manner of doing the work; it was at that time confessedly imperfect. The whole aim of that editorial was to direct attention to, and excite a spirit of inquiry concerning the matter, and these objects were accomplished. Dr. Slayton had about as much to do with either of the notices as Dr. Hunter. We generally make our own remarks in regard to matters of the kind, without any special dictation, and give just the impressions we may have at the time; always standing in readiness to get out of a false position whenever we are so unfortunate as to be in one.

"The cunningly devised notice," for the immense benefit of the inventor, would hardly be seen without very acute vision.

### VULCANITE BASE FOR TEETH.—DR. ROBERTS, N. Y.

Our readers have been familiar through our advertising columns, with this valuable invention, and they will be interested to learn, we doubt not, the position of affairs in regard to the matter, as it has elicited no little attention for some time past.

We have just learned from our correspondent in New York, that at the last term of the United States Circuit Court, held in that city, and just closed, that in a suit brought against Dr. Roberts, by the Goodyear interest, a motion was noticed for an injunction to restrain him from using, in the manufacture of plates for artificial teeth, Vulcanized Gutta Percha, and from selling such plates to others, when manufactured. But the defence made by Dr. Roberts, to such motion, was so complete, that the counsel of the Goodyear party abandoned his motion without argument.

As this matter is exciting not a little interest in our profession, we shall give in our next number, a copy of Goodyear's Patent of 1851, and his re-issue of 1858, that all may judge of the claims made in each.

### THE TRIP TO NIAGARA.

Arrrangements are about being completed, by which the fare will be reduced very much —to probably half fare—from Cincinnati to Niagara, to all those going to the "American Dental Convention." All persons South and West of this, and indeed all who may come to Cincinnati can avail themselves of this arrangement. All persons coming and wishing to embrace this arrangement, will call upon John T. Toland, No. 38 West Fourth street, for all special information in regard to this arrangement.

It is expected now, that every body will go and take his wife. We hope the ladies of the profession will appreciate our labor on their behalf. T.

### DENTAL DEPOT.—SUTTON G. RAYNOR.

By reference to the advertising sheet, it will be perceived that this enterprising firm, is doing business with its accustomed energy and ability. In the dental furnishing line, they manifest a disposition not to be excelled. Tneir aim is to furnish all articles in the dental line, of superior quality, and they accomplish it. This after all is the great thing with the dentist. His instruments and materials should be of the best quality obtainable ; the price paid is only a secondary consideration. In regard to prices this firm is as accommodating as any other. They are emphatically go-ahead men, and will not be excelled.

---

The Druggist.—We have before us "The Druggist," a monthly newspaper, published by C. S. Williams, 194 Walnut Street, Cincinnati. The first number was issued in April. It is for the benefit of the Druggists, and is,furnished at a merely nominal price.

Each number will contain at least eight pages of original and select matter. The markets will be revised every month. By such means the Druggists and Physicians, and all others in any way connected with the business, or interested in it will keep posted up. None such can well afford to be without it

---

### A PROPOSITION.

The D. M. C. of the Ohio Dental College proposes to see to the safe delivery and transfer of all baggage, to and from all Railroads, Steamboats and Hotels, belonging to those Dentists who make Cincinnati their starting point for the coming Convention ; also when there, (in Convention,) to attend to their wants, the same manner he did when the Convention was in Cincinnati. In return for the above, all he asks is, that his fare be paid to and from Cincinnati, and Niagara Falls by the above dentists, which, from the number understood to be going, will be about 50 cts. each. All in favor of the above will say *I!!* and notify Dr. Taft of the fact before the 1st of August.

